# A Cross-Sectional Study of Barriers in Prevention of Anemia in Pregnancy

**DOI:** 10.7759/cureus.12802

**Published:** 2021-01-20

**Authors:** Archana Mishra, Sheeba Marwah, Pragati Divedi, Rupali Dewan, Himani Ahluwalia

**Affiliations:** 1 Department of Obstetrics and Gynecology, Vardhman Mahavir Medical College and Safdarjung Hospital, New Delhi, IND; 2 Department of Obstetrics and Gynecology, Rama Medical College and Hospital, Hapur, IND; 3 Department of Physiology, Vardhman Mahavir Medical College and Safdarjung Hospital, New Delhi, IND

**Keywords:** anaemia, pregnancy, iron deficiency, antenatal care, non -compliance, iron intolerance, chronic diseases, late registration

## Abstract

Introduction

Anemia in pregnancy is a significant health challenge in India and other developing countries. Various health programs aiming anemia prevention are existing in India for many decades. Despite that, anemia affects more than half of pregnant women. Our objective for performing this study was to evaluate the barriers in the prevention of anemia and to evaluate the perceptions and practices of anemic women towards their condition.

Methods

A cross-sectional questionnaire-based study, including 210 anemic women, was conducted in a tertiary care center in Delhi, India. In-depth interviews were conducted with 50 participants.

Results

Our important observations were that anemia was more prevalent in multigravida, and 43.80% of anemic patients were not taking iron supplements at the time of diagnosis. Chronic diseases were associated with 28.2% (n=59) of anemic women. Only 19% (n=40) of women sought antenatal care in the first and second trimester; the rest all booked themselves in the third trimester only. Twenty-two percent (n=48) of women reached our hospital after 36 weeks. Ignorance to anemia symptoms and the importance of consistent intake of the oral iron supplements was seen in 35.2% (n=74). One hundred and sixty-five (74.8%) women accepted that healthcare provider had informed them about iron-rich and high protein diet, but only 47.1% (n=98) actually made dietary modifications. Only 9.5% (n=20) of women were consistent in iron intake. Side effects of iron were reported by 30% (n=64) of women, and 15% (n=32) were intolerant to oral iron. Non-availability, change of residence, and forgetfulness were the main reasons behind non-compliance to oral iron.

Conclusion

We emphasize motivation for early registration, regular antenatal care, adequate iron supplement supply, and persistent counseling to ensure compliance to iron supplements by the antenatal care provider. Behavior-changing communication is needed at a societal level for antenatal mothers and their families aiming to prevent anemia in pregnancy instead of focussing on treatment.

## Introduction

Anemia in pregnancy is a significant health challenge in developing countries, including India. It is a condition that is defined as the hemoglobin percentage below the expected levels of 11 gm% [1]. According to WHO, 56% of pregnant women in the developing world suffer from anemia. Anemia is directly responsible for 20% of maternal mortality and indirectly contributes to another 20% [2]. National Family Health Survey in 2019 revealed that 50% of reproductive age women are anemic in India [3]. Many factors aggravate this problem in pregnancy, such as physiological hemodilution, nutritional iron deficiency, protein and vitamin deficiency, underlying inflammatory conditions, etc. Anemic women are 2-4 times more prone to many complications, such as the increased risk of cardiac diseases, decreased work productivity, preterm delivery, postpartum hemorrhage, and infections. Newborn of anemic mothers suffer from low birth weight, poor cognitive development, and low immunity [4].

Iron deficiency is the most common cause of anemia in pregnancy, but it never occurs alone - it is usually accompanied by a deficiency of other nutrients [5]. To combat this problem, the National Nutritional Anemia Prophylaxis Programme (NNAPP) was launched in India in 1970. NNAPP, along with Integrated Child Development Services (ICDS) scheme, is the largest national program for promoting mother and child health and their development [6]. Both programs had three important strategies: the promotion of regular consumption of foods rich in iron, provisions of iron and folate supplements in the form of tablets to the high-risk groups, especially pregnant women, and the identification and treatment of severely anemic cases. There is a provision of 60 mg of elemental iron with 0.5 mg of folic acid to all the pregnant women for at least 100 days of pregnancy [6]. This program has an estimated 20 million adult beneficiaries. Despite all these efforts for 48 years, the prevalence of anemia still ranges from 33% to 89% among pregnant women in different regions of the country [7]. The high magnitude of anemia in pregnancy with associated grave health consequences highlights the need to identify the barriers to anemia prevention. The purpose of this study was to evaluate the barriers to anemia prevention and evaluate the perceptions and practices of anemic women towards their condition. 

## Materials and methods

A cross-sectional study was performed in a tertiary care center in North India from November 2019 to November 2020. Ethical clearance was obtained from the Institutional Ethics Committee. After informed consent, we recruited the anemic pregnant women. Inclusion criteria:

1. Anemia in pregnancy documented by hemoglobin less than 11 gm% either by complete blood picture or by Sahle’s acid hematin method of hemoglobin estimation.

2. Clinical symptoms and signs of anemia.

Exclusion criteria involved those who had anemia due to acute antepartum hemorrhage.

Purposive sampling was done for all the subjects. All the participants were interviewed using a pre-structured questionnaire (see Appendix). This questionnaire was pretested on 25 participants for ease of understanding. The questionnaire had five sections. The first section consists of questions regarding participants' demographic characteristics such as age, religion, socioeconomic status, etc. The second section has questions regarding obstetric history, trimester history, and past history of the participant. The third section included questions assessing the knowledge of participants regarding anemia and supplements. The fourth section has questions evaluating the attitude of participants for antenatal care and the prevention of anemia. The fifth section included questions to reveal the actual practices of the participants. At the end of the interview, queries from the pregnant women were addressed, and counseling regarding a balanced diet and compliance with iron supplements was done. Every fourth participant was chosen for an in-depth interview where questions were asked about the perceptions of antenatal care and prevention of anemia. Participants explained in their own language their reasons for non-compliance with iron supplements.

Ethical clearance was taken from the Ethics Committee of our institution.

Most of the results were described in percentages. Analysis of variance (ANOVA) test was applied for the analysis of variables.

## Results

A total of 210 anemic antenatal women were recruited for the study who were either booked or registered in our antenatal outpatient department (OPD). Only 42 (19.70%) pregnant women were considered as booked as they had at least three previous visits to any health care facility. One hundred and sixty (58%) women had only one visit at any peripheral center from where they were referred to our center. Forty-eight women (22.85%) never visited any healthcare facility, and they directly reached our hospital. Table 1 shows the sociodemographic profile of the participants. Table 2 depicts the distribution of antenatal mothers as per obstetric history and booking status. Table 3 describes the past history, and Table 4 reveals the knowledge and attitudes of anemic patients. Table 5 reveals the actual practices of these patients, and Table 6 illustrates the common reasons for non-compliance.

**Table 1:** Sociodemographic profile of anemic mothers

Characteristics	Number (percentage), n=210
Age in years	
<25	87 (41.42%)
25-30	63 (30%)
30-45	60 (28.57%)
Religion	
Hindu	180 (85.71%)
Muslim	26 (12.38% )
Christian	2 (0.95%)
Sikh	2 (0.95%)
Socioeconomic status	
Upper middle class	12 (5.7%)
Lower middle class	34 (16.19%)
Lower socioeconomic status	164 (78.0%)

**Table 2:** Distribution of anemic mothers according to their obstetric history and booking status

Obstetric history	Number (percentage), n=210
Primigravida	53 (25.23%)
Multigravida	157 (74.76%)
Registration at gestational week	
<14	22 (10.47%)
14-28	20 (9.25%)
28-32	80 (38.09%)
32-36	60 (28.57%)
36- 40	48 (22.85%)

**Table 3:** Distribution of anemic mothers according to their past history

No.	Past history	Number (percentage), n=210
1	Anemia in previous pregnancy in multigravida	140 (66.66%)
2	Chronic disease	59 (28.09%)
3	Menorrhagia	40 (19.04 %)
4	Hemoglobinopathy	30 (14.28%)
5	Malaria	20 (9.52%)
6	Hypothyroidism	19 (9.04%)
7	Worms	14 (6.7%)
8	Blood transfusion	12 (5.7%)
9	Hematuria	7 (3.33%)
10	Piles/Fissure	4 (1.9%)

**Table 4:** Description of knowledge and attitudes of anemic mothers about anemia prevention

Knowledge/attitudes	Number (percentage), n=210
Knowledge	
Requirement of iron increases during pregnancy	74 (35.2%)
Know about iron supplement tablets	157 (74.8%)
Adequate information on iron-rich food and balanced diet given by provider	127 (60.4%)
Know that iron/calcium tablets provided by government free of cost	74 (35.2%)
Attitude	
Considered excessive edema in pregnancy is a sign of anemia	101 (41.8%)
Agreed that weight gain is essential for pregnancy	115 (54.7%)
Considered pallor as a sign of anemia requiring treatment	101 (48.1 %)
Considered easy fatiguability and breathlessness alarming	44 (20.9%)

**Table 5:** Practices of anemic mothers

Practices	Number (percentage), n=210
Adequate weight gain during pregnancy	95 (45.2%)
Increased nutritional intake iron/protein/folic acid/calcium	130 (61.9%)
Got hemoglobin estimation in first trimester	20 (9.5%)
Consistent in intake of daily dose of Iron	20 (9.5 %)
Iron salt taken is iron sulfate	92 (43.8%)
Changed iron salt in case of intolerance	26 (12.5%)
Accepted intravenous iron sucrose advised for oral Iron intolerance	27 (12.5%)
Refused iron sucrose advised for oral iron intolerance	5 (2.3%)
Taken protein supplement	99 (47.1%)

**Table 6:** Reasons for non-compliance 190 participants were non-compliant for iron supplements.

Reason	Number (percentage), n=190
Change of place	70 (36.8%)
Not enough supply	40 (21.05%)
Forgetfulness	38 (20%)
Gastritis	12 (5.7%)
Constipation	10 (4.7%)
Other	20 (9.52%)

We conducted in-depth interviews with 50 participants, where they described their difficulties in their own language. Here we present common observations. The concept of regular antenatal care was missing in most of the participants. Most participants believed that there is no need to go to the hospital in early pregnancy and that they need to visit the hospital on the fifth or sixth month of pregnancy for an ultrasound. One participant noted: *"I was sent to my native place at five months of pregnancy as there is no family support here". *Similar statements were made by other participants as well. Most of the women accepted that they are unable to buy iron supplements. One of the participants uttered that *"tablets from government supply finished before the next visit and these are too expensive to buy"*. Twenty women left iron supplements due to gastric intolerance, but they did not mention it to their doctor. 

## Discussion

Prevention and treatment of anemia in pregnancy require coordinated actions among multiple stakeholders and partners. Family and pregnant women are the most important of them. Perceptions of pregnant women towards anemia guide the success of the anemia prevention program. 

In the present study, multigravida patients were more prone to developing anemia than primigravida patients, which is contrary to other studies published from different regions of India [8, 9]. Surprisingly 140 (64.81%) multigravida patients suffered from anemia in previous pregnancy also. In fact, 12 had a history of blood transfusion too. Despite this, no measures were taken by them to prevent anemia in the index pregnancy. It shows the low perceived risk in the target group. The same observation was made in another study from Africa that primigravida listens and complies with medical advice, but as the age and burden of routine chores increases, the negligence of a woman towards her own health also increases [10].

In our study, 85.7% of anemic women were Hindu, which is similar to other studies from India, which has shown the similar prevalence in Hindu pregnant women as 88.8% and 89.9% of all anemic women [9]. In the present study, we found that 28% of the anemic women were suffering from chronic illness either in pregnancy or in the recent past. Evidence from the literature supports the presence of functional iron deficiency in chronic illnesses. There is a relative deficiency of iron in the blood, and most of the iron is stored in the form of ferritin. This protective mechanism of the body is supposed to slow the progression of chronic illness as iron has a pro-oxidant role [11]. Such observation highlights the importance of proper history taking and simultaneous treatment of chronic illness. Such patients often require a higher dose of iron supplemented with other micronutrients. Thirty women were positive for thalassemia minor trait, which was not detected previously.

Only 9.5% of women booked themselves for proper antenatal care each in the first trimester and in the second trimester. All of these women were advised for hemoglobin estimation, but even patients who booked early also were not regular in subsequent visits, investigations, and management. A maximum number of women came to our hospital in the third trimester only. This was the major reason why the patient did not get timely counseling and interventions to prevent anemia.

The service provider also has an important role in ensuring the adequate intake of iron supplements by pregnant women [12]. In the present study, only 35% of women knew about the need for iron supplementation in pregnancy, and it is being provided free of cost by the government supply. Even after contact with the medical facility, only 46.7% of women had actually taken iron supplementation, and only 47.1% of women had taken a high protein diet. The difference was seen between knowledge and actual practices of pregnant women, which was the main reason for nutritional anemia. Despite knowledge about weight gain, only 54.7% of women had adequate weight gain. Only 40% of women started taking a protein-rich and iron-rich diet. Low educational status, low income, and limited access to nutritious diet also play an important role in anemia [13].

Side effects of oral iron were found only in 64 (30.47%) women. Thirty-two (15.23 %) women were prescribed other salt of iron and were comfortable with that. Rest 32 (15.23%) women were intolerant to most of the types of oral iron preparations and were suggested intravenous iron sucrose. Only 27 women complied with it. Some of the lacunae were found on the part of care providers also. Follow-up questioning confirming compliance of iron and high protein diet is frequently missed by providers. In one more study, it was reported that non-compliance increases three-folds in case of side effects, but prior counseling by health care providers does improve compliance [12]. 

We observed gross unawareness regarding symptoms and signs of anemia in pregnant women. Almost three-fourths of women do not consider edema as a sign of anemia or any other pathology in pregnancy. Only 19% considered pallor an abnormal finding and only 27% considered that fatigue needs medical attention. Early booking and regular antenatal care can easily help such women and prevent serious complications and the need for blood transfusion.

Fifty-eight percent of women had nausea and vomiting in the first and second trimesters, so total calorie and nutrients intake was much less than required in that crucial period. Considering nausea and vomiting normal symptoms of pregnancy, they did not seek medical advice. Untreated nausea and vomiting may be one important reason for inadequate food intake and the inability to start iron. Only 11% of women complaint about newly developed nausea and vomiting after ingestion of Iron.

Consistency in the intake of iron was found in hardly 9.5% of women. The rest of the women were skipping iron tablets at trivial excuses. Most of the women who claim to consume iron also had taken hardly 30-40 tablets of iron only. Other studies also support that non-compliance with an iron supplement is the major reason behind anemia in pregnancy [14, 15]. Important reasons given behind the non-compliance were a change of places during pregnancy, forgetfulness, and not enough supply from the government hospital.

One important observation of our study was that a large cohort of these women was of wives of migrant laborers who spend a major part of their pregnancy at their native place outside Delhi and visit government hospital only in the late third trimester, which is too late for any intervention to prevent anemia. We emphasize the awareness regarding early antenatal booking and compliance for anemia preventive measures in this particular target group.

The second vital observation was that antenatal visits in the second trimester and early third trimester are widely spaced, but the supply of iron to individuals at every visit is much lesser. Women are supposed to buy the rest of the drugs or get them from a government dispensary nearby. Both the options did not comply, and none of the women was able to take “suggested 100“ tablets in total duration of pregnancy. In other studies from the developing world revealed similar reasons for non-compliance such as gastrointestinal side effects, inadequate supply of tablets (including limited resources to purchase tablets), inadequate counseling of woman by healthcare providers, poor utilization of prenatal health-care services, myths and fear about the tablets and community beliefs, attitudes and practices that affect pregnant women's perception regarding tablet use [16].

Early registration is recommended by WHO and guidelines provided by the Ministry of Health and Family Welfare a policy in our hospital we encourage accredited social health activist (ASHA) workers from surrounding areas for early registration of pregnancy. We also use a mass media approach by playing videos regarding early registration and antenatal care in our OPD waiting area. 

## Conclusions

We emphasize on the grass-root level empowerment of anemia prophylaxis program beginning from primary health centers. The coverage, quality, and efficiency of the NNACP need to be improved. Women at high risk for anemia should be detected in early pregnancy. The aim of antenatal care should be to prevent anemia rather than treatment of already anemic pregnant women. The mindset of the general population, especially women of childbearing age, must be changed. Mass media approach addressing this issue could be useful. All the providers, including doctors, midwives, auxiliary nurse-midwives (ANM), and even ASHA, are recommended to motivate the pregnant women for early registration and full compliance with iron preparation and a balanced diet. Issues like non-availability and intolerance to oral iron should be promptly addressed and rectified.

## Appendices

Study questionnaire

- Name:

- OPD No:         

- Age:

- Occupation:

- Education:     

- Socioeconomic status:

- Parity formula:       Gravida ……….. Para………. Abortion……….. No. of Living Children………..

- Booked at gestational age :

- Referral:  Self/ Some other hospital:

- Booked or registered only:

- HB% at the time survey    

11- 10 gm%

10-7 gm%

<7 gm%

<4 gm%

Past history:  

- Any chronic disease :

- Anemia in previous pregnancy:

- Blood transfusion in previous pregnancy:

- Menorrhagia prior to pregnancy:

- Hypothyroidism:

- Weakness/ fatigue prior to pregnancy:

- Hemoglobinopathy/ Malaria/Kala Ajar/Hematuria/Piles/Fissure/ Bleeding Per Rectum/Worm infestation

Knowledge:

- Do you know iron/calcium tablets are provided by government free of cost? Yes/No

- You understands the need of iron supplementation during pregnancy: Yes/No

- You were provided information on iron-rich food: Yes/No

- You were advised to take oral iron supplementation: Yes/No

- You were told to take iron before meals and not along-with food: Yes/No

- You were told about balanced diet: Yes/No

- You were aware of high protein diet/calcium diet: Yes/No

Attitudes:

- Do you consider edema as normal feature of pregnancy? Yes/No/Don’t know

- Do you consider weight gain is required during pregnancy? Yes/No/Don’t know

- Do you consider pallor as normal feature of pregnancy? Yes/No/Don’t know

- Do you consider weakness and fatigue as normal feature of pregnancy? Yes/No/Don’t know

Practices:

- Weight gain in pregnancy: Adequate/ Inadequate

- Nutritional intake of iron, protein, folic acid and calcium as calculated by three days recall method: Adequate /Inadequate

- Hemoglobin estimation done in first trimester: Yes/No

- At what POG  iron and calcium supplementation started:

- Amount of iron tablets taken after fourteen weeks: no of tablets/no of days taken

- Daily dose of iron: 100mg/less

- What iron preparation were you taking? FESO4 /other

- Did you take Iron sucrose when prescribed? Yes/No

- Did you take protein supplement? Yes/No

- Reasons for inability to comply with iron supplement

Points which were discussed in in-depth interviews:

- Cross questions to confirm iron Intake

- Iron preparation - compliant/non-compliant

- Reason for noncompliance discussed in detail

1) Forgetfulness

2) Change of places

3) Not enough supply in hospital

4) Constipation

5) Gastritis

6) Participant don’t think Iron is important

7) Excessive nausea& vomiting during pregnancy

- Socio/cultural barrier for balanced diet

- Any myths regarding Iron preparations

- Is her family supportive for dietary changes in pregnancy?

If the participant was found non-compliant then these questions have to be asked

- Had she informed the health professionals about the difficulties?

- Had she tried to change the iron preparation to improve compliance?

- Had she received additional advice to overcome the problems responsible for discontinuation?
